# ESC Cardio-Oncology Guidelines: A Triumph—But Are We Overscreening?

**DOI:** 10.1016/j.jaccao.2022.10.008

**Published:** 2022-12-06

**Authors:** Ronald M. Witteles, Sunil A. Reddy

**Affiliations:** a Division of Cardiovascular Medicine, Stanford University School of Medicine, Stanford, California, USA; b Division of Oncology, Stanford University School of Medicine, Stanford, California, USA

**Keywords:** anthracycline, anti-angiogenic therapy, biomarkers, cancer survivorship, echocardiography, guidelines, her2 therapy, immunotherapy, screening, tyrosine kinase inhibitor

It is difficult to assign a precise date to the birth of the field of cardio-oncology. Although cardiac effects of anthracyclines have been known for decades, the field as we now know it truly emerged in the early to mid-2000s, largely in response to recognized cardiac toxicities from trastuzumab and vascular endothelial growth factor (VEGF) inhibitors. Since that time, the field has exploded, mirroring the exponential growth of new cancer therapeutics; toxicities grew from a focus on left ventricular dysfunction/heart failure to include vascular, electrophysiologic, pericardial, thrombotic, and valvular toxicities.

How does one measure milestones of the maturation of the field? The very existence and growth of this journal is one; 15 to 20 years ago, it would have been hard to conceive that an entire *JACC* journal could be devoted to the field. In that context, the simultaneous publication and presentation of the new European Society of Cardiology (ESC) Cardio-Oncology guidelines^1^ to a packed auditorium should be viewed as nothing less than a triumph—and as further evidence that the field of cardio-oncology has hit the mainstream.

And a triumph these guidelines are. The numbers themselves are astounding: 30 international experts from 13 countries collaborated to put together a 133-page document (plus an additional 45-page supplement) with 837 references. The committee aimed to do no less than to cover the entire field of cardio-oncology, including toxicities across every major class of cancer therapeutics, primary and secondary prevention strategies, diagnostic and therapeutic pathways—and more! The guidelines include 82 tables and 48 figures; the tables reviewing drug–drug and drug–food interactions alone would be a monumental achievement, and yet there is so much information in the guidelines that these are found in the supplemental material.

The writing and recommendations are clear, the tables/figures are direct and concise, and the comprehensive nature of the document is truly impressive. The overriding theme—that the goal of cardio-oncology is to maximize the safe administration of cancer therapies rather than to limit treatment with effective cancer therapies—is a laudable one. If health care providers or cancer patients have a question about anything cardio-oncology related, they will almost certainly find it addressed. This is even more impressive when one considers that these are the first such guidelines—meaning that all of the content had to be created from scratch, rather than building upon previous efforts. That is a tremendous achievement, and one which every member of the task force should take great pride in.

Where might the guideline authors have taken a different tact? It largely depends on the manner in which one views the purpose of guidelines. Should guidelines be more conservative in their recommendations—restricting their recommended diagnostic and treatment strategies to those areas with a solid evidence-base? Or, should guidelines be more aspirational—going out on a limb to make recommendations where the evidence base may not be there, but recommending practices that committee members believe are more likely than not to be a good idea. The ESC Cardio-Oncology guidelines clearly take the latter approach.

But is it a good idea? One of the most important roles for guidelines is to set the standard of care for a field. If guidelines are seen as overreaching, they run the risk of being ignored. A potential litmus test for any guideline recommendation can start with the question, “What percentage of providers currently meet what is being recommended?” If the number is <50%, most providers are by definition providing care below the “standard-of-care,” a concept that is in and of itself an oxymoron. There are potential legal implications as well, with liability a real risk if a bad patient outcome occurs and a provider is seen as not having followed the “official guidelines” in diagnosis or management. For the ESC Cardio-Oncology guidelines, our sense is that the percentage of providers who would currently be meeting the guideline recommendations is extremely small. Is that okay? Are providers in the field that much behind the evidence, or does it mean that the guidelines have overreached? Our view is that it is the latter.

Consider the document’s recommendations for biomarker screening:

• The guidelines recommend baseline cardiac troponin (cTn) and natriuretic peptide (NP) measurements for *all patients* treated with agents that have the potential to cause cancer therapy–related cardiovascular toxicity. This is despite the fact that, as the guideline authors concede, “the literature on the use of biomarkers for cancer therapy–related cardiovascular toxicity risk stratification before cancer therapy is limited.”^1^
• NP and cTn monitoring is recommended at baseline, every 3 months, and 12 months after therapy even in *low- and moderate-risk* HER2+ early breast cancer patients.^1^
• NP monitoring is recommended at baseline and every 4 months for the first year in moderate-risk patients receiving a VEGF inhibitor.^1^
• NP measurements are recommended every cycle for the first 6 cycles even in *low- to moderate-risk patients* receiving bortezomib, despite a lack of evidence that bortezomib treatment even predisposes to a higher risk of heart failure.^1^^,^^2^
• Cardiac biomarkers are recommended 12 months after completion of cancer therapy in asymptomatic moderate-risk patients (Class IIa) and even in asymptomatic low-risk patients (Class IIb).

How many oncologists meet these recommendations? Is there reasonable evidence to guide any of this?

Consider the troponin screening recommendations for immune checkpoint inhibitor (ICI) therapy. The guidelines recommend cTn measurements at baseline, before doses 2, 3, 4, and every 3 doses thereafter.^1^ Our institution has more experience than most with cTn screening for ICI myocarditis, having performed one of the early studies testing such an approach.^3^ Although we detected 3 early cases of myocarditis (1.4% of patients screened), we also learned the challenges of such a screening method—not the least of which were both a high rate of false positives and the need to provide staffing to safely triage elevated troponin results whenever they resulted. Even as a group that advocates the potential of cTn screening, we recognize its challenges/limitations and do not believe the evidence basis is there to begin to make such a broad recommendation for the entire ICI-treated population.

Next, consider the imaging guidelines.

• For low-risk patients receiving HER2-targeted therapy, left ventricular ejection fraction and global longitudinal strain assessments are recommended before the start of therapy, every 3 months while on treatment, and within 12 months after completing treatment.^1^ This translates to 6 echocardiograms for screening in *low-risk patients*, despite clear evidence from clinical trials that the rate of heart failure in the low-risk non–anthracycline-treated population is small, and possibly no higher than in the general population.^4^ Even for low-risk patients with metastatic disease, echocardiograms are recommended every 3 months during the first year and every 6 months thereafter. This recommendation furthermore does not differentiate between trastuzumab and other therapies such as pertuzumab or trastuzumab emtansine, which trials have demonstrated have even lower rates of left ventricular ejection fraction declines.^5^
• Most patients treated with VEGF inhibitors are recommended to have echocardiograms every 3 to 4 months in the first year and every 6 to 12 months thereafter, even though the document notes “evidence is weak.”^1^ Should we not have a higher standard to make such a broad recommendation?
• Echocardiograms are recommended every 3 cycles for even low-risk patients receiving carfilzomib—even though the heart failure associated with carfilzomib is primarily diastolic and not well-characterized by echocardiography.^6^
• Echocardiograms are recommended for patients with previous cardiovascular disease receiving fluoropyrimidines^1^—despite the fact that fluoropyrimidine cardiotoxicity is almost exclusively angina/vasospasm, rather than changes in cardiac structure/function that would be detected by echocardiography.
• Post-therapy echocardiograms within 12 months of therapy completion are recommended for all patients treated with potentially cardiotoxic treatments^1^—even in asymptomatic, low-risk patients. We know of no evidence basis to support this recommendation.

What about the echocardiograms themselves? Seventeen different metrics are recommended for *every* cancer patient having an echocardiogram,^1^ including such unvalidated assessments as right ventricular free wall longitudinal strain. Considering the number of patients who receive cancer therapies and the number of assessments recommended for each study, echocardiogram labs would be overwhelmed if these guidelines were ever followed. Where is the evidence supporting that each echocardiogram necessitates all of these measurements being acquired/interpreted, with the extra time, expense, and resources required?

All of this proposed screening would result in real costs. If the guidelines were to be followed as written, the monetary costs to patients and the health care system from non–evidence-supported imaging, laboratory testing, and therapeutic interventions would be enormous. One must further consider the time and emotional costs to patients, who may get labeled with new “diseases” that they do not truly have. In addition, all of this testing adds to an overburdened health care system, as recently highlighted by the COVID pandemic, which may lead to delays in more strongly indicated testing for other patients.

Broad recommendations are made for medical interventions, beyond what the current evidence supports. Angiotensin-converting enzyme inhibitors, angiotensin receptor blockers, and beta-blockers are recommended for primary prevention in high-risk patients receiving anthracyclines and/or anti-HER2 therapies, despite a series of clinical trial results which range from unimpressive to frankly negative.^7^, ^8^, ^9^ Consider statins, which were recommended in the guidelines for patients at high cardiotoxicity risk on the basis of extremely limited evidence. Eight days before the guidelines were released, the first trial to properly assess their role was published in patients treated with anthracyclines—revealing no cardioprotection for atorvastatin compared with placebo in a 2-year randomized trial.^10^ Although there was no way for the writing group to take this result into account at the time of the creation of the guidelines, it illustrates the challenges when guidelines overreach the evidence—a large number of the recommendations are likely to not stand the test of time. This is an essential certainty when only 2.6% of the 272 recommendations met an evidence grade of A, with more than 75% earning the lowest evidence grade of C.^1^

Although we wish a more restrained path had been chosen when it comes to many of the recommendations, the ESC guidelines nevertheless represent a seminal moment in our field of cardio-oncology. They are a true triumph, and the writing committee who worked tirelessly to put them together deserve our respect, our praise, and our congratulations.

## Funding Support and Author Disclosures

Dr. Witteles has participated in advisory boards for Pfizer, Alnylam, Ionis, Bridgebio, NovoNordisk, Janssen, Intellia, and AstraZeneca. Dr Reddy has reported that he has no relationships relevant to the contents of this paper to disclose.
